# Health system performance assessment in small countries: The case study of Latvia

**DOI:** 10.1002/hpm.2803

**Published:** 2019-05-15

**Authors:** Guido Noto, Ilaria Corazza, Kristīne Kļaviņa, Jana Lepiksone, Sabina Nuti

**Affiliations:** ^1^ Management--> and Health Laboratory, Institute of Management Scuola Superiore Sant'Anna Pisa Italy; ^2^ Human Resource Development Division Ministry of Health Riga Latvia; ^3^ Centre of Disease Prevention and Control Riga Latvia

**Keywords:** benchmarking, evidence‐based management, health system performance assessment, performance measurement, small countries

## Abstract

Managing the complexity that characterizes health systems requires sophisticated performance assessment information to support the decision‐making processes of healthcare stakeholders at various levels. Accordingly, in the past few decades, many countries have designed and implemented health system performance assessment (HSPA) programmes. Literature and practice agree on the key features that performance measurement in health should have, namely, multidimensionality, evidence‐based data collection, systematic benchmarking of results, shared design, transparent disclosure, and timeliness.

Nevertheless, the specific characteristics of different countries may pose challenges in the implementation of such programmes. In the case of small countries, many of these challenges are common and related to their inherent characteristics, eg, small populations, small volumes of activity for certain treatments, and lack of benchmarks.

Through the development of the case study of Latvia, this paper aims at discussing the challenges and opportunities for assessing health system performance in a small country.

As a result, for each of the performance measurement features identified by the literature, the authors discuss the issues emerging when adopting them in Latvia and set out the potential solutions that have been designed during the development of the case study.

## 1 INTRODUCTION

Healthcare systems are characterized by an intrinsic complexity derived from governance fragmentation as well as uncertainty, pluralism, and a multidisciplinary environment.^1^, ^2^, ^3^ Health systems embrace resources, organizations, financing mechanisms, and governance models that culminate in the delivery of health services to the population they serve; they are the results of decisions taken by many stakeholders at different levels to face a broad spectrum of problems.^3^, ^4^

Given such a level of complexity, it is challenging to embrace health systems in a holistic view—ie, to understand their overall functioning.^4^ Health system performance assessment (HSPA) programmes are developed to address this need.^5^ According to the definition provided by the World Health Organization (WHO), HSPA aims to be a country‐owned, participatory process that allows the health system to be assessed as a whole using a limited number of quantitative and qualitative performance indicators and that should be linked to national health strategies.^6^

Assessing the performance of health systems is important for a number of diversified reasons, which are often complementary and could even be mutually reinforcing.^7^, ^8^, ^9^ Above all, the key goals of HSPA are related to the improvement of the health system performance and population health. According to a report of the European Union,^9^ the specific goals of HSPAs may range from the support to decision makers at various levels—eg, to design strategies, implement incentives' schemes, and improve quality of care—to accountability purposes and patient empowerment.

A seminal model to assess health services performance was developed by Donabedian.^10^ This model distinguished three main domains according to an instrumental view of performance management,^11^ namely, structure, process, and outcome. Evolutions of the Donabedian work were carried out by scholars belonging to different fields, such as public health, healthcare management, and performance management, by means of scientific and working papers.^12^, ^13^, ^14^, ^15^, ^16^, ^17^, ^18^, ^19^ The result, explored in detail in the theoretical background section of this article, is a set of features that should characterize performance management and evaluation processes in the health sector, namely, *multidimensionality*, *evidence‐based data collection*, *systematic benchmarking of results*, *shared design*, *transparent disclosure*, and *timeliness*.

Even though literature and practice agree on the characteristics that should characterize HSPA processes, the design and implementation of these systems may present several challenges when tailored to specific realities. This happens to be the case of small countries, ie, countries having a population under three million inhabitants.^20^

These challenges are related to the inner characteristics of small states, which refer to vulnerabilities associated with small size, lack of economies of scale, limited capacity, and significant exposure to external economic shocks.^20^, ^21^ Other issues refer specifically to the measuring and monitoring of performance metrics, eg, statistical significance of results and lack of benchmarks.^22^

Based on the theoretical background related to performance management studies in the health sector, the ultimate purpose of this study is to answer the following research question:

RQ: How to design and implement HSPA in small countries?

In order to pursue this goal, the present article focuses on the case of a small European country, namely, Latvia. This represents an interesting case since, starting in October 2017, Latvia has undertaken a process of design, development, and implementation of an HSPA.

The paper is structured as follows. Section 2 outlines the theoretical background applied to this research, ie, performance management and assessment in health systems. Section 3 focuses on the method adopted to develop the Latvian case study. Then, a section concerning the results of the research is developed. Lastly, discussion and conclusions tackle the main challenges and opportunities emerging through the development of the case study and related to HSPA in small countries.

## 2 THEORETICAL BACKGROUND: PERFORMANCE MANAGEMENT AND ASSESSMENT IN HEALTH SYSTEMS

The introduction of performance measurement and management practices in the health sector has been characterized by different phases.^19^ The first exercises have been developed according to the New Public Management (NPM) paradigm of the 1980s, which, with regards to performance assessment, promoted the use of private sector practices and values and, above all, the focus on financial measures and volumes of services provided.^23^, ^24^ NPM was the major public sector reform approach that many western nations adopted during the period between the early 1980s and the mid‐1990s so as to respond to a concern with government failures and a belief in the efficiency of markets. This reform was indeed aimed at solving the shortcomings with regard to efficiency and effectiveness, which are intrinsic in the traditional bureaucratic approach characterizing public administration in western countries during the 20th century.^23^, ^25^ Although overcoming some of the limits of the bureaucratic approach, the first performance management systems designed in this period—because of their focus on financial measures and volumes of services provided—limited the ability of healthcare stakeholders to assess performance according to the public value paradigm. This, in the last few decades, has become the reference paradigm for public administrations.^25^, ^26^ Public value is a multidimensional construct that primarily results from public sector performance.^27^, ^28^ In healthcare, public value has been defined as the relationship between outcomes and resources^29^ from a population‐based perspective.^30^, ^31^ The underlying concept of the public value paradigm is the adoption of a systemic approach that takes into consideration the interests of several stakeholders in the healthcare system and embraces a population‐based perspective, ie, the ability of the healthcare system to provide care to the people that could benefit most from it.^31^

The need to redefine the design of performance management systems according to the public value paradigm resulted in a push from governments and scholars to identify the key features that should characterize performance management and assessment of health systems.

As previously mentioned, these features have been explored by previous literature and summarized by Nuti et al^19^ in the following six items: *multidimensionality*, *evidence‐based data collection*, *systematic benchmarking of results*, *shared design*, *transparent disclosure*, and *timeliness*.

*Multidimensionality* refers to the need of taking into account the different dimensions of health systems when assessing their performance. As previously mentioned, traditionally, performance measures in the public sector have been primarily focused on financial results.^13^, ^32^, ^33^ The strong focus on the financial performance of performance management system generation resulted in various limits mainly related to dysfunctional performance results.^19^, ^25^ In order to overcome these limits, performance management systems started to be designed including multiple performance dimensions.^33^ The healthcare sector has not escaped this performance management system's evolution. The dimensions generally considered by HSPAs could vary from a system to another based on the information needs that should be produced so as to respond to the ultimate scope for which the HSPA has been put in place. As for example, among the key dimensions that could be considered, the WHO suggests effectiveness, efficiency, accessibility, patient‐centredness, safety, and equity.

*Evidence‐based data collection* refers to the need to inform decision makers through data and information based on real evidence. Evidence‐based management derives from evidence‐based medicine—ie, the conscientious, explicit, and judicious use of current best evidence in making decisions about the care of individual patients^34^—and it is based on the assumption that managers should ground their judgement and practice on rational, transparent, and rigorous evidence that could help them explore and evaluate the pros and cons of alternatives and that they should be informed by relevant, robust academic research and literature reviews.^35^, ^36^ Evidence emerges in healthcare as the keystone for informing decision making at various levels. At the micro level, evidence could support in solving frequent conflicts among physicians' different experiences and opinions about the most appropriate clinical practice to be adopted in certain cases. At the organizational level, managers could use evidence to design their strategies and actions. Finally, at the macro level, policy makers should invest in administrative health database research to extract evidence to identify those policies that might work better in their health system^35^ and to set priorities and targets.^37^, ^38^

*Systematic benchmarking* of results has gained in the last decades a pivotal role when measuring performance in the public sector. According to Francis and Holloway,^39^ “benchmarking has been a method for identifying aspects of an organization's activity that could be more efficient and/or effective by comparison with other relevant organizations' performance.” However, this is only one of the possible definitions of benchmarking that the literature has produced in the lasts decades.^40^, ^41^, ^42^, ^43^ Especially in the public sector, where the market does not represent a real reference for assessing performance, benchmarking has turned into a fundamental tool in the modernization of public services,^44^ where it is associated with four positive outcomes. These outcomes^45^ are (a) the identification of good practices from organizations run at both the national and international level either in the private or in the public sector, (b) the process of improvement against competitors in the same sector that goes through monitoring, (c) the identification of best suppliers, and (d) the process of improvement in central policy making and service delivery. In the realm of the services offered in the public sector, benchmarking has been widely extended as a management practice also to the health sector. According to Nuti et al,^46^ in the health sector, benchmarking among providers and among geographic areas could represent a standard in order to shift from monitoring to evaluation—ie, the systematic determination of a unit merit, worth, and significance using criteria governed by a set of standards. Benchmarking is also recognized to be an incentive for entities belonging to the same field to move along a harmonization process, which in turn implies a diminishing in the performance variations.^44^ In the healthcare sector, enhancing benchmarking of protocols and measures is positively associated with a reduction of unwarranted variation in performance between organizations and geographical areas, where unwarranted variation could be defined as the variation in health services that cannot be explained by patient illness and preference but is mainly explained by health system performance.^47^, ^48^

*Shared design* refers to the need for involving the wide set of key stakeholders of the health system when designing an HSPA. The involvement of key players of the health systems is considered pivotal to ensure the high quality of the HSPA process and to create ownership and legitimation among stakeholders and the wider community.^17^ To do that, according to a literature review conducted by Leggat et al,^14^ it is important that the performance management model incorporates the fundamental values, attitudes, and information needs of the users of the performance indicators, eg, citizens, patients, professionals, and the entities participating to the governance of the health system. The involvement of health professionals is considered particularly important in order to provide insights and suggestions (eg, new indicators and revision of existing indicators) in a continuous fine‐tuning process.^14^, ^49^

*Transparent disclosure* is related to the decision to make HSPA results available to an external audience or not. Although it seems like a trivial aspect, the publishing of results can have important effects on healthcare organizations and professionals, especially when it is linked to governance strategies. These effects are mainly related to reputation mechanisms^50^ and to the rise of potential performance distortions—eg, “gaming”—if the HSPA and the performance targets linked to it are not properly designed.^51^ In synthesis, literature agrees that transparent disclosure of performance‐related data is a positive driver as long as it is implemented so as to leverage on reputation levers and it takes into account the governance system in which it is applied as a whole in order to avoid unintended consequences.^50^, ^52^

*Timeliness* refers to the need for making HSPA results available to inform decision makers as soon as possible.^53^, ^54^ This is important for two main reasons. First of all, it allows decision makers to react promptly to the emergence of problems shown by data. As for example, HSPA that provide data with a 2‐year delay cannot be used by decision makers to make decisions since the environment and the performance may have been significantly changed. Second, organizations, units, and professionals whose performance is assessed according to non‐timely computed indicators may perceive the whole process as unfair, thus losing trust in the HSPA.

While literature agrees on the need to design HSPAs that complies with the features outlined above, real experiences often face issues in addressing them. This happens for a variety of reasons mainly related to the social and environmental characteristics of the health system involved. In the case of small countries, many of these issues are related to their dimensions, population, and density. This paper aims at advancing knowledge on how small countries may deal with the design of HSPAs and overcome some of the technical issues that may emerge because of their dimensions.

As reported in the introduction of this work, the following paragraph illustrates the method employed by this research so as to investigate challenges and opportunities in developing HSPA processes in small countries.

## 3 METHOD

This paper is based on a case study.^55^ In particular, the research develops the Latvian HSPA case. The choice to focus on this country relies on three main reasons. First of all, with 1.93 million inhabitants, Latvia can be considered as a small country (see Central Statistical Bureau of Latvia data). Second, the international comparison shows that Latvia performs critically in some key area of the health system.^56^ Lastly, the country has recently started taking part in a process aimed to develop and implement an HSPA framework, and the authors have been actively engaged in it. This project has been founded by the Structural Reform Support Service (SRSS) of the European Commission.

This article is based on the action research developed within the abovementioned project.

According to what was prescribed by the main literature on performance measurement in health,^14^, ^19^ the design of the HSPA involved all of the key institutions and stakeholders operating in the Latvian Health System. These belong to various categories that range from government institutions—eg, the Ministry of Health (MoH), Centre for Disease Prevention and Control (CDPC), National Health Service (NHS), and Health Inspectorate (HI)—to several hospitals and universities.

This joint, fine‐tuning, and systematic process was carried out by organizing a series of meetings among the parties involved in the project. Overall, a number of 16 meetings were organized and put in place. These meetings took place either physically (ie, training sessions, workshops, and study visits) or through the use of videoconferences.

As a result, in between December 2018 and February 2019, a final version of the Latvian HSPA and the related action plan (ie, the implementation plan) was delivered and approved by the project's Steering Committee. In the next sections, the results of this process are reported and discussed.

## 4 THE LATVIAN HSPA CASE STUDY

### 4.1 The Latvian Health System

The Latvian Health System is a general, tax‐financed healthcare provision system that is embedded within a social insurance institutional structure and is characterized by a purchaser‐provider split with a mix of public and private providers. Despite the numerous attempts made in the last decades in order to make the health system more efficient and effective, some criticalities still remain. To mention a few, although life expectancy has been increasing in recent years, it remains to be lowest among the Baltic and Nordic countries,^56^ at an average of 74.9 years in 2016. Besides the high mortality rate due to diseases of the circulatory system, infant mortality also remains slightly above the EU 28 average—3.7 deaths per 1000 live births—though it has fallen substantially^56^ since 1980. Even in the perspective of mental health, problems arise, given that suicide rates are higher than in any western European country, constituting the fifth most common cause of death.^57^

Today in Latvia, health services are regulated by the central government, which operates through the funding of the NHS and other national institutes (among which we find the CDPC, the HI, the State Agency of Medicines [SAM], and the State Emergency Medical Service [SEMS]). The NHS is a state‐run organization under the MoH. It is the central institution responsible for pooling health funds, purchasing health services, and contracting and paying healthcare providers (a mix of public and private providers). Second and tertiary care is organized and delivered directly at the state level, while primary care and ambulatory care is usually delivered by local governments (municipalities) and private providers. Local governments usually have their own budgets, and their main task is to assure geographical accessibility and engage in healthy lifestyles promotion.

### 4.2 The Latvian HSPA framework

The ultimate goal of the Latvian HSPA framework is to support the improvement of the health system performance with respect to the three main objectives of a public health system, namely, equity, quality of care, and financial sustainability.

On the basis of the previous experience of Latvia “health care reform performance scorecard” approved through Order no. 394 of the Cabinet of Ministers, 7 August 2012, and built upon the Donabedian conceptual model,^10^ the HSPA framework for Latvia has been characterized by four main domains, which are *Structures*, *Processes*, *Outcomes*, and *Patient Experience*, organized in multiple key dimensions, namely, *Financial Resources*, *Human Resources*, *Equipment*, *Efficiency*, *Quality*, *Safety*, *Prevention*, *Demand Management*, *Equity*, *Accessibility*, and *Health Status*. Moreover, in order to take into account the national health strategies already in place, four care pathways, which are *Mental Health*, *Oncology*, *Cardiovascular Diseases*, and *Maternal and Child Health*, have been identified within the domains of *Processes* and *Outcomes*.

**Figure 1:**
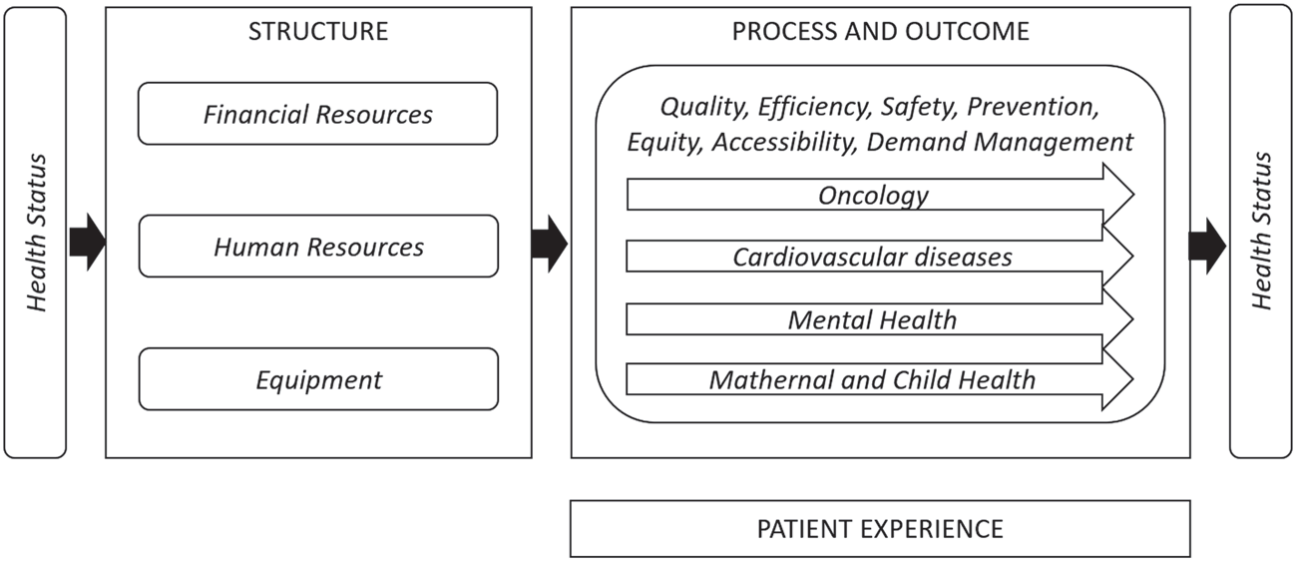
The Latvian health system performance assessment (HSPA) framework

Figure 1 shows the final Latvian HSPA framework as described above.

In order to populate the HSPA framework with performance measures, a selection of indicators has been made. The selection process has been conducted by (a) taking into account literature and international experience review, (b) involving different categories of key stakeholders in the system, and (c) considering the indicators and data already available in Latvia. The indicators selected are ratios whose numerators, denominators, and computation have been peer‐reviewed by a group of experts.

Each indicator was classified into a specific domain, one or multiple dimensions (since some indicators could support the assessment of different performance dimensions), and, when appropriate, into a specific pathway (the full list of indicator can be made available upon request).

To date, the framework consists of an overall number of 191 indicators classified in the domains, dimensions, and pathways as portrayed in Table 1. Today, the *Patient Experience* domain has not been populated by indicators yet because of the lack of available data. A pilot project is currently under development in order to collect data so as to cover this domain in the next years.

**Table 1:** Population of domains, dimensions, and pathways

DOMAINS	Structure	23	Process	80	Outcome	88	Total	191
DIMENSIONS	Financial Resources	10					Financial Resources	10
	Human Resources	10					Human Resources	10
	Equipment	3					Equipment	3
			Prevention	10			Prevention	10
			Efficiency	15			Efficiency	15
			Accessibility	5			Accessibility	5
			Quality	20	Quality	57	Quality	77
			Safety	6	Safety	19	Safety	25
			Equity	7	Equity	1	Equity	8
			Health Status	6	Health Status	27	Health Status	33
			Demand Management	24	Demand Management	4	Demand Management	28
PATHWAYS			Cardiovascular diseases	2	Cardiovascular diseases	17	Cardiovascular diseases	19
			Maternal and child path	10	Maternal and child path	11	Maternal and child path	21
			Mental Health	3	Mental Health	4	Mental Health	7
			Oncology	8	Oncology	32	Oncology	40

As previously maintained, some indicators belong to multiple dimensions since they can be used to describe the performance of different dimensions—as for example, the indicator “Percentage of GP patients contacted by a GP in the 30 days after discharge” belongs to the dimensions *Efficiency*, because it measures the ability of primary care to deliver the visits due in time, and *Demand Management*, because a good primary care service may allow avoiding hospitalizations.

Among the 191 indicators identified, 82 indicators have been classified as *evaluation indicators*—indicators whose results are going to be evaluated according to a desired trend/value and whose results could be attributed to the activities of the healthcare organizations operating within the different geographic areas (regions and municipalities).

**Figure 2:**
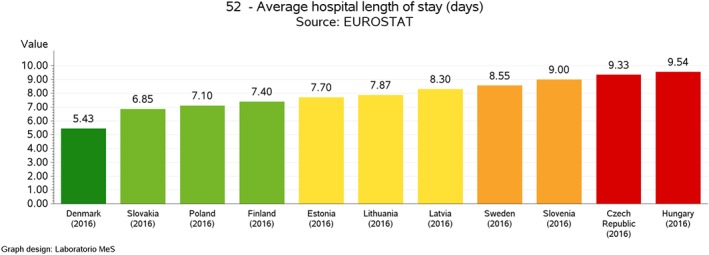
Average hospital length of stay, intercountry comparison [Colour figure can be viewed at http://wileyonlinelibrary.com]

**Figure 3:**
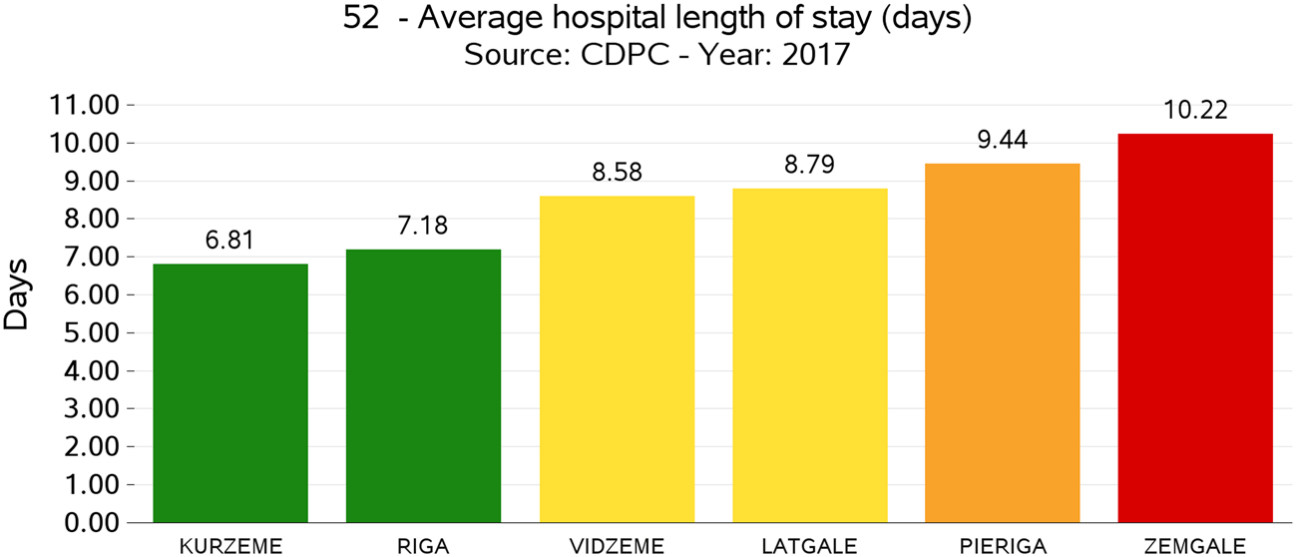
Average hospital length of stay, interregional comparison [Colour figure can be viewed at http://wileyonlinelibrary.com]

**Figure 4:**
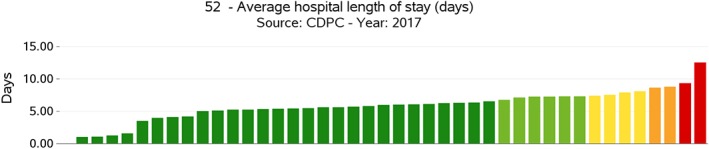
Average hospital length of stay, provider comparison (anonymised) [Colour figure can be viewed at http://wileyonlinelibrary.com]

The evaluation process is carried out according to the existence of gold standards or through international benchmarking. For each indicator, based on the statistical distribution of values undertaken by the different units measured (eg, countries), five performance intervals (or evaluation bands) are defined, ranging from a very good performance to a very bad one. According to the type of indicator, evaluation bands can be either “increasing” or “decreasing.” An indicator is considered to be increasing when a high evaluation of it is associated with a positive performance, while in the case of a decreasing indicator, a high evaluation of the indicator is associated with negative performance. Moreover, in terms of graphical representation of the results, the evaluation bands computed for each indicator are associated to a colour scale, where the red colour indicates a very poor performance while the dark green colour indicates an outstanding performance (see Figures 2, 3, and 4).

The definition of a set of evaluation indicators is especially important in the perspective of identifying and managing performance at various levels. In particular, the collection, computation, and evaluation of indicators were conducted according to three benchmarking tiers. In fact, despite the small geographical dimensions, Latvia presents a certain level of internal fragmentation, since it is articulated respectively into six statistical regions, 119 administrative municipalities, and 1893 healthcare providers (including general practitioners). Because of that, the choice has been made to conduct the benchmarking analysis not only at the intercountry level but also at the geographical‐area level (regions and municipalities) and at the provider level. At the intercountry level, Latvian experts selected a panel of 12 European countries to benchmark with, namely, Lithuania, Estonia, Slovenia, Slovakia, Poland, Czech Republic, the Netherlands, Hungary, Finland, Sweden, Portugal, and Denmark. These countries were selected based on their geographic, demographic, political, and socio‐economic characteristics. At the local level, Latvian performance was computed according to the subdivision in statistical regions. At the provider level, the HSPA considered each provider delivering health services financed in total or in part by the Latvian NHS.

In procedural terms, when data are available for each level, performance intervals are defined first at the higher level, that is, the intercountry one, and then are applied to the lower ones, which are the regional/municipal levels or the provider one. This procedure avoids the onset of cases when best performers at the national level are positively evaluated even though poorly performing when compared at the international level and vice‐versa.

As for example, Figures 2 and 3 show the results of one of the indicators of the Latvian HSPA framework, which is an indicator belonging to the *Processes* domain and characterizing the dimension of *Efficiency*, the *Average Hospital Length of Stay*, expressed in absolute terms of the number of days spent in the hospital. Even though Latvia shows an average performance when compared with other countries (see Figure 2), at the domestic level, there is a significant variation among its statistical regions (Figure 3), where two of them show a poor performance even when compared with the average value of the other countries in Figure 2. This internal variation is even more evident when comparing performance at the provider level (see Figure 4).

In order to provide a clear and aggregate representation of the regional health system performance results to policy makers and other stakeholders, the Latvia HSPA adopted as visual representation the dartboard graph. This way of representing the performance of the health system was originally developed by the Management and Health Laboratory of the Scuola Superiore Sant'Anna to assess the performance of various Italian regions and was then adopted by different experiences at the international level (see, for instance, the 2017 Country Health Profile by the European Commission, the Organisation for Economic Co‐operation and Development (OECD), the New Zealand dashboard developed by the Health Quality & Safety Commission New Zealand, the National Health Performance Authority of Australia, and the Dartmouth Institute). The dartboard is a circle graph in which excellent performances are reported in a dark‐green area positioned in the centre while worse performances are progressively reported in the peripheral area of the graph whose colour shifts from dark green to light green, yellow, orange, and red.

**Figure 5:**
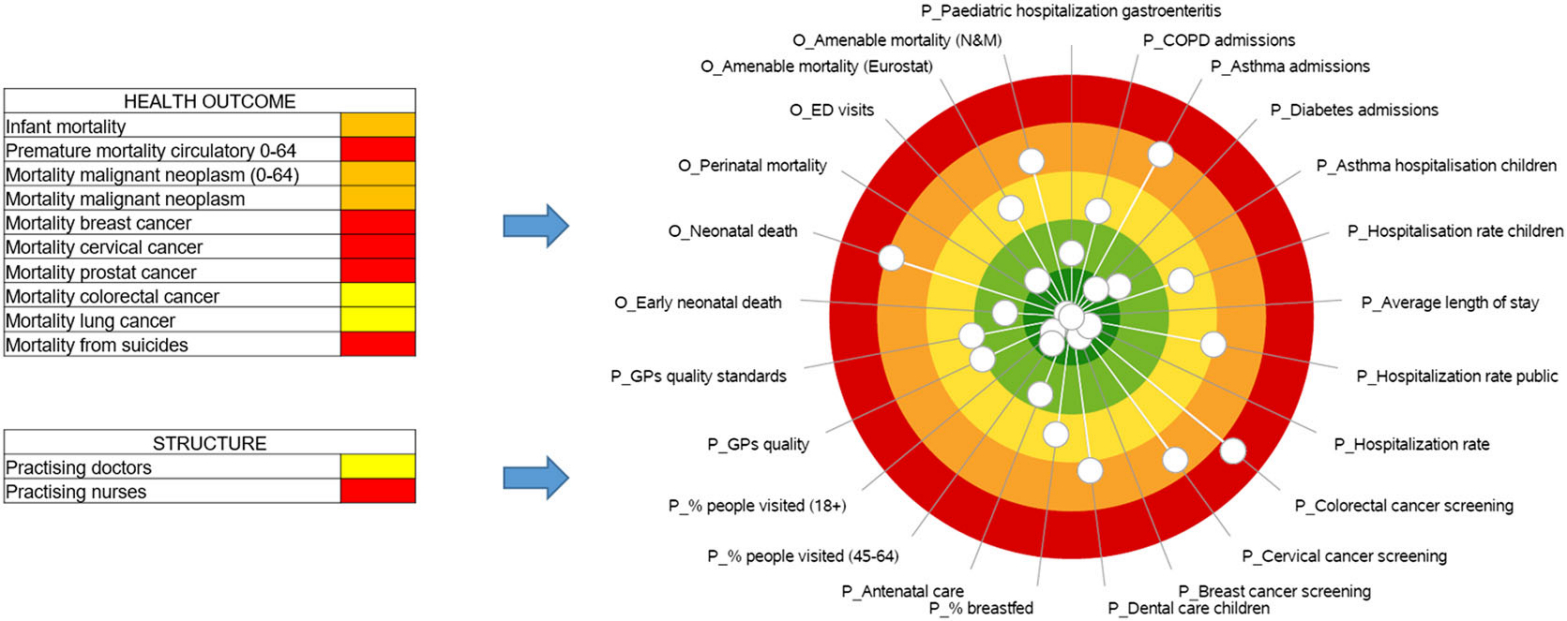
An example of the overall representation of a Latvian region performance [Colour figure can be viewed at http://wileyonlinelibrary.com]

This was applied at the three levels identified to assess performance, ie, country level, regional level, and provider level. Figure 5 shows the 2017 evaluation of a Latvian region performance.

**Figure 6:**
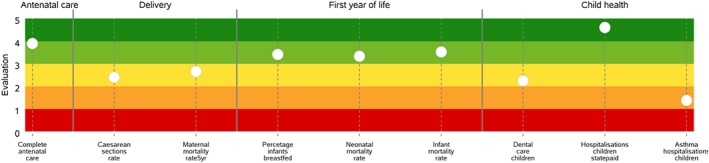
An example of the *Maternal and Child Health* pathway performance of a Latvian region [Colour figure can be viewed at http://wileyonlinelibrary.com]

On the basis of the specific information needs of the users, the HSPA designed for Latvia could produce details related to the performance of specific dimensions or pathways. As for example, Table 2 shows the indicators belonging to the *Maternal and Child Health* pathway, while Figure 6 displays its performance—ie, the evaluated indicators available—in one of the Latvian regions.

**Table 2:** The set of indicators populating the *Maternal and Child Health* pathway

Phase	Code	Indicator	Domain	Dimension
Antenatal care	19	Complete antenatal care	Process	Quality & Safety
	63	Births without obstetric intervention	Outcome	Quality
	64	Caesarean sections per 1000 live births	Process	Quality & Demand Management
	65	Obstetric trauma during vaginal delivery with instruments	Outcome	Quality & Safety
	66	Obstetric trauma during vaginal delivery without instrument	Outcome	Quality & Safety
	95	Rate of caesarean sections by Robson classification in 1st group	Process	Demand Management
	96	Rate of caesarean sections by Robson classification in 2nd group	Process	Demand Management
	146	Fetal mortality rate	Outcome	Quality & Safety
	147	Maternal death rate per 100000 live births, 5 years average	Outcome	Quality & Safety
	148	Maternal death rate per 100000 live births	Outcome	Quality & Safety
First year of life	20	Percentage of infants breastfed at age 6 months	Process	Quality & Safety
	142	Infant mortality rate (per 1000 live births)	Outcome	Quality & Safety
	143	Perinatal mortality, per 1000 live births	Outcome	Quality & Safety
	144	Neonatal death, per 1000 live births	Outcome	Quality & Safety
	145	Early neonatal death, per 1000 live births	Outcome	Quality & Safety
Child health	18	Percentage of children who receive preventive examination	Process	Efficiency & Accessibility
	21	Dental care of children: percentage of children, who had a visit to a dental care specialist during the last year	Process	Efficiency & Accessibility
	61	Hospitalization of children (0‐17 years), state‐paid service	Process	Demand Management
	62	Asthma hospitalization for children (2‐17 years)	Process	Demand Management
	94	Paediatric hospitalization rate for gastroenteritis	Process	Demand Management
	177	Measles, mumps and rubella immunization coverage for second dose (%)	Process	Prevention

The graphical representation adopted to represent the pathway performance is the “stave.”^19^ The stave replicates the patients' pathway along with the clinical processes and services that characterize the care delivery focusing on the strengths and weaknesses that characterize the healthcare service delivery. Moreover, the stave includes indicators that could be related to the activity of different providers (eg, primary care, ambulatory care, and hospitals). This supports the accountability of the health system as a network of stakeholder, which are called to address the population needs as a whole.

## 5 DISCUSSION

The Latvian experience of the design process of an HSPA represents an interesting case study to explore the key challenges and opportunities related to performance assessment of the health system in small countries.

As reported in the previous section, the Latvian HSPA was developed and defined according to the key features identified by the literature and practice, which are reported in the theoretical background of this article.

Concerning the first feature, ie, *multi‐dimensionality*, the Latvian HSPA responded to it by identifying, within the four domains defined according to Donabedian seminal work,^10^ 11 dimensions and four key pathways. The dimensions identified support the possibility of focusing on certain characteristics of the health systems such as efficiency and accessibility. The pathways are cross‐dimensional and allow HSPA users to focus on the key clinical pathway identified by the policy makers as key priorities.

The *evidence‐based data collection* prescription was followed by focusing on the collection of data coming from reliable sources of information and building indicators according to literature suggestions and international experiences. With respect to the small dimensions of the country, the presence of small volumes of the activities or episodes of care measured at the municipal and provider levels implied the need to design innovative solutions to provide correct information to policy makers.

Dealing with the presence of small volumes at the municipal and provider level is a direct and unavoidable consequence of fragmenting scarcely populated geographic areas in multiple subunits of analysis. If, on the one hand, such a process of fragmentation is necessary in order to allow the benchmarking of different territorial units and to assess internal variation,^47^, ^48^ on the other one, for some indicators, it may be problematic in terms of volumes of activity and episode of care, because under these conditions, even one single record in the data flow (eg, an episode of care) can have a great impact on final indicator results. In order to tackle this aspect, the Latvian experience explored two solutions. The first option was applied at the territorial level (municipalities and regions) and refers to the adoption of the rolling averages methodology used in many small European countries based on the indications offered by the Small Countries Health Information Network (SCHIN)^22^ of the WHO. It is possible to apply such a methodology whenever data over multiple years are available for the indicators considered, making the use of moving averages possible. In particular, the SCHIN^22^ suggests adopting the rolling average for indicators that have less than 10 records (at the denominator) and to consider a time frame of 3 or 5 years, depending on the degree of annual fluctuation of the indicator value. The second option, applied at the provider level, refers to the definition of evaluation criteria (ie, the criteria according to which the evaluation bands are defined) that takes into account the production volumes (ie, the number of treatments for a specific type of intervention). This criterion implies the need to exclude from the evaluation process the providers that do not report a sufficient volume of data for the result to be statistically significant by assigning them an average evaluation.

As reported in the results, *systematic benchmarking of results* was designed and implemented at three levels: intercountry, territorial, and provider one.

At the intercountry level, comparing results with those of other European countries with similar characteristics turned out to be an interesting opportunity for Latvia, which, being a small country, may lack best practices to benchmark with in some performance dimensions. The European Union, and in general terms the international comparison, could thus provide the opportunity to put in place a learning community in which small countries will be able to benchmark among themselves and with bigger countries or with geographical areas of bigger countries.

At the regional level, during the process of designing the HSPA, it emerged that, even though the benchmarking of performance results highlights interesting insights, there are no administrative governance levels nor political representatives, which have direct levers to implement actions based on the evidence produced by the performance indicators. The Latvian regions were indeed created for statistical purposes, and, moreover, there is no evidence demonstrating that these represent the optimal subdivision of the country in geographic areas for health policies implementation. The representation of the results based on geographical areas opened an interesting debate between the stakeholders involved in the design of the HSPA. This debate was focused, on the one hand, on the opportunity to provide performance indicators results at the regional level to the national government, which is called to design and implement policies aimed at dealing with the related equity issues. On the other hand, it was discussed whether to explore the opportunity to reform the governance structure of the Latvian health system in order to create new entities or governance levels with the power to intervene on the planning and regulation of the health services related to each geographical area. Anyhow, all the stakeholders agreed that benchmarking at the regional level sheds light on some key issues related to the avoidable geographical variation that characterizes the Latvian health system. Before the results of the HSPA, these issues were only perceived by some of the stakeholders, but scarce evidence has been produced to assess the related phenomena.

The *shared design* feature was addressed by involving in the process of definition of the HSPA framework and the related indicators a broad range of stakeholders. The stakeholders selected comprehended all the entities directly involved the governance of the health system (Ministry of Health, NHS, HI, CDPC, SAM, and SEMS), the management of a selected set of providers, universities, and some representative professionals. A specific meeting was also organized to discuss with professionals the computation of some key outcome indicators (eg, 30‐day Acute Myocardical Infarction mortality). In small countries, this process could be eased by the reduced complexity of the health system governance structure, ie, the presence of less governance levels compared with bigger countries.

For what concerns the topic of the *transparent disclosure* of results, it was agreed that the HSPA results are to be made publicly available starting from 2019. With regards to 2018 data, the disclosure will be exclusively directed to the key stakeholders involved in the design process and the managers and key professionals of the healthcare providers evaluated.

Lastly, *timeliness* has been ensured by designing the HSPA so that data and information are provided within 6 months from the closing of the year considered. This would allow policy makers to use the information produced in the policy cycle, ie, to define priorities and set objectives of the health system and the healthcare providers.

## 6 CONCLUSIONS

According to the authors' opinions, the Latvian case study represents a useful experience to be shared with scholars and professionals involved in the design, implementation, and use of HSPAs.

The key challenges that small countries should be prepared to deal with when designing and implementing HSPA processes are related to (a) the significance of some metrics that take into account a limited number of activities or episodes of care and (b) the need to support internal benchmarking practices with some external reference so as to evaluate key performance indicators. Concerning the first issue, the solution implemented in Latvia was related to the need to compute indicators over multiple years or to exclude from the evaluation of indicators some entities reporting low values at the denominator (even though showing the related data). The second issue was addressed by implementing a three‐level benchmarking practice across countries, geographical areas, and healthcare providers and using as a reference for the evaluation, when possible, the intercountry comparison.

The main limit of this research is related to the generalization of results since the article is based on a single case study, and the results, such as the solutions adopted to deal with the small dimensions of the country analysed, are necessarily linked to the country characteristics, environment, and governance setting. Moreover, these represent some of the possible solutions that could be implemented when assessing performance in small countries or even in small regions within countries. However, the authors do not exclude the possibility of further developing the lesson learned through the Latvian case study by focusing on other cases of small countries.

## References

[1] Plsek PE , Greenhalgh T . Complexity science: The challenge of complexity in health care. BMJ Br Med J. 2001;323(7313):625‐628. 11557716 10.1136/bmj.323.7313.625 PMC1121189

[2] Lemieux‐Charles L , McGuire W , Champagne F , Barnsley J , Cole D , Sicotte C . The use of multilevel performance indicators in managing performance in health care organizations. Manag Decis. 2003;41(8):760‐770. 10.1108/00251740310496279

[3] Smith RD , Hanson K . Health Systems in Low‐ and Middle‐Income Countries: An Economic and Policy Perspective. Oxford: Oxford Sch; 2012.

[4] Paoli F , Schmidt I , Wigzell O , Ryś A . An EU approach to health system performance assessment: building trust and learning from each other. Health Policy (New York). 2019:in press;123:403‐407. 10.1016/j.healthpol.2019.02.004 30777300

[5] World Health Organization (WHO) . *The world health report 2000. Health systems: improving performance*; 2000.

[6] World Health Organization (WHO) . *Pathways to health system performance assessment: a manual to conducting health system performance assessment at national or sub‐national level*; 2012.

[7] Smith PC , Mossialos E , Leatherman S , Papanicolas I . Performance Measurement for Health System Improvement: Experiences, Challenges and Prospects. Cambridge: Cambridge University Press European O; 2009.

[8] Papanicolas I , Smith PC . Health System Performance Comparison: An Agenda for Policy, Information and Research. Maidenhead: Open University Press; 2013.

[9] European Commission . *Reporting and Communication of HSPA findings* 2017.

[10] Donabedian A . The quality of care. How can it be assessed? JAMA. 1988;260(12):1743‐1748. 3045356 10.1001/jama.260.12.1743

[11] Bianchi C . Improving performance and fostering accountability in the public sector through system dynamics modelling: From an ‘external' to an ‘internal' perspective. Syst Res Behav Sci. 2010;27(4):361‐384. 10.1002/sres.1038.

[12] Murray CJL , Frenk J . Theme Papers A framework for assessing the performance of health systems. 2000. PMC2560787 10916909

[13] Ballantine J , Brignall S , Modell S . Performance measurement and management in public health services: a comparison of U.K. and Swedish practice. Manag Account Res. 1998;9(1):71‐94.

[14] Leggat SG , Narin L , Lemieux‐Charles L , et al. A review of organisational performance assessment in healthcare. Health Serv Manage Res. 1998;11:3‐18. 10.1177/095148489801100102 10178369

[15] Aidemark L . The meaning of balanced scorecard in the health care organization. Financ Account Manag. 2001;17(1):23‐40.

[16] Arah OA , Westert GP , Hurst J , Klazinga NS . A conceptual framework for the OECD Health Care Quality Indicators Project. Int J Qual Heal care J Int Soc Qual Heal Care. 2006;18(Suppl 1):5‐13. 10.1093/intqhc/mzl024 16954510

[17] European Commission . *So What? Strategies across Europe to assess quality of care* 2016.

[18] Nuti S , Vola F , Bonini A , Vainieri M . Making governance work in the health care sector: evidence from a ‘natural experiment' in Italy. Health Econ Policy Law. 2016;11(1):17‐38. 10.1017/S1744133115000067 25819303 PMC4697307

[19] Nuti S , Noto G , Vola F , Vainieri M . Let's play the patients music: a new generation of performance measurement. Manag Decis. 2018;56(10):2252‐2272. 10.1108/MD-09-2017-0907

[20] Azzopardi‐Muscat N , Funk T , Buttigieg SC , Grech KE , Brand H . Policy challenges and reforms in small EU member state health systems: a narrative literature review. Eur J Public Health. 2016;26(6):916‐922. 10.1093/eurpub/ckw091 27335326

[21] Thorhallsson B . The size of states in the European Union: Theoretical and conceptual perspectives. J Eur Integr. 2006;28(1):7‐31.

[22] World Health Organization (WHO) . *Third Meeting of the Focal Points of the Small Countries Health Information Network (SCHIN)* Copenhagen 2017.

[23] Hood C . A public management for all seasons? Public Adm. 1991;69(1):3‐19.

[24] Brignall S , Modell S . An institutional perspective on performance measurement and management in the ‘new public sector'. Manag Account Res. 2000;11(3):281‐306. 10.1006/mare.2000.0136.

[25] O'Flynn J . From new public management to public value: paradigmatic change and managerial implications. Aust J pub Admin. 2007;66(3):353‐366. 10.1111/j.1467-8500.2007.00545.x

[26] Cuganesan S , Jacobs K , Lacey D . Public value management, measurement and reporting article information. Public Value Manag Meas Report. 2014;3:21‐42.

[27] Moore MH . Creating Public Value: Strategic Management in Government. Harvard, MA: Harvard University Press; 1995.

[28] Bryson JM , Crosby BC , Bloomberg L . Public Value Governance: Moving beyond traditional public administration and the new public management. Public Adm Rev. 2014;74(4):445‐456. 10.1111/puar.12238

[29] Porter ME . What is value in health care? N Engl J Med. 2010;363(26):2477‐2481. 21142528 10.1056/NEJMp1011024

[30] Gray M , El Turabi A . Optimising the value of interventions for populations. BMJ Br Med J. 2012;345(sep17 1):e6192 10.1136/bmj.e6192. 22988306

[31] Gray M , Airoldi M , Bevan G , McCulloch P . Deriving optimal value from each system. J R Soc Med. 2017;110(7):283‐286. 28537103 10.1177/0141076817711090 PMC5524260

[32] Chua WF , Preston A . Worrying about accounting in health care. Acc, Audit Accountability J. 1994;7(3):4‐17.

[33] Bititci U , Garengo P , Dorfler V , Nudurupati S , Dörfler V , Nudurupati S . Performance measurement: challenges for tomorrow *. Int J Manag Rev. 2012;14(3):305‐327. 10.1111/j.1468-2370.2011.00318.x

[34] Sackett DL , Rosenberg WMC , Gray JAM , Haynes RB , Richardson WS . Evidence based medicine: what it is and what it isn' t. BMJ Br Med J. 1996;312:71. 8555924 10.1136/bmj.312.7023.71 PMC2349778

[35] Aloini D , Cannavacciuolo L , Gitto S , Lettieri E , Malighetti P , Visintin F . Evidence‐based management for performance improvement in healthcare. Manag Decis. 2018;56(10):2063‐2068.

[36] Lomas J , Brown A . Research and advice giving: a functional view of evidence‐informed policy advice in a Canadian Ministry of Health. Milbank Q. 2009;87(4):903‐926. 20021590 10.1111/j.1468-0009.2009.00583.x PMC2888020

[37] Vainieri M , Vola F , Gomez Soriano G , Nuti S . How to set challenging goals and conduct fair evaluation in regional public health systems. insights from Valencia and Tuscany regions. Health Policy (New York). 2016;120(11):1270‐1278. 10.1016/j.healthpol.2016.09.011 28029416

[38] Nuti S , Vainieri M , Vola F . Priorities and targets: supporting target‐setting in healthcare. Public Money Manag. 2017;37(4):277‐284. 10.1080/09540962.2017.1295728

[39] Francis G , Holloway J . What have we learned ? Themes from the literature on best‐ practice benchmarking. Int J Manag Rev. 2007;9(3):171‐189. 10.1111/j.1468-2370.2007.00204.x

[40] Zairi M , Youssef MA . A review of key publications on benchmarking: part I. Benchmarking Qual Mgmt Tech. 1995;2(1):65‐72. 10.1108/14635779510081652

[41] Cox A , Thompson I . On the appropriateness of benchmarking. J Gen Manag. 1998;23:1‐20.

[42] Dorsch JJ , Yasin MM . A framework for benchmarking in the public sector: literature review and directions for future research. Int J Public Sect Manag. 1998;11(2/3):91‐115.

[43] Anderson K , McAdam R . A critique of benchmarking and performance measurement: lead or lag? Benchmarking an Int J. 2004;11(5):465‐483.

[44] van Helden GJ , Tillema S . In search of a benchmarking theory for the public sector. Financ Account Manag. 2005;21(3):337‐362.

[45] Bowerman M , Ball A , Francis G . Benchmarking as a tool for the modernisation of local government. Financ Account Manag. 2001;17(4):321‐329.

[46] Nuti S , Seghieri C , Vainieri M . Assessing the effectiveness of a performance evaluation system in the public health care sector: some novel evidence from the Tuscany region experience. J Manag Gov. 2013;17(1):59‐69. 10.1007/s10997-012-9218-5

[47] Wennberg JE , Gittelson A . Small area variations in health care delivery: a population‐based health information system can guide planning and regulatory decision making. Science. 1973;182(4117):1102‐1108. 4750608 10.1126/science.182.4117.1102

[48] Nuti S , Vainieri M . Strategies and tools to manage variation in regional governance systems In: Johnson A, Stuckel TA, eds. Medical Practice Variations. US: Springer; 2016:433‐457.

[49] Nuti S , Bini B , Ruggieri TG , Piaggesi A , Ricci L . Bridging the gap between theory and practice in integrated care: the case of the diabetic foot pathway in Tuscany. Int J Integr Care. 2016;16(2):1‐14. 10.5334/ijic.1991 PMC5356204 29042842

[50] Bevan G , Evans A , Nuti S . Reputations count: why benchmarking performance is improving health care across the world ☆. Health Econ Policy Law. 2018;14:141‐161. in press. 10.1017/S1744133117000561 29547363

[51] Bevan G , Hood C . What's measured is what matters: targets and gaming in the English public health care system. Public Adm. 2006;84(3):517‐538. 10.1111/j.1467-9299.2006.00600.x

[52] Mannion D , Davies HTO . Reporting health care performance: learning from the past, prospects for the future. J Eval Clin Pract. 2002;8(2):215‐228. 12180369 10.1046/j.1365-2753.2002.00331.x

[53] Davies HTO , Lampel J . Trust in performance indicators ? Qual Health Care. 1998;7(3):159‐162. 10185142 10.1136/qshc.7.3.159 PMC2483599

[54] Wadmann S , Johanses S , Lind A , Okkels Birk H , Hoeyer K . Analytical perspectives on performance‐based management: an outline of theoretical assumptions in the existing literature. Health Econ Policy Law. 2013;8(4):511‐527. 10.1017/S174413311300011X. 23506797

[55] Yin RK . The abridged version of case study research: design and method. Thousand Oaks, CA: Sage; 1998.

[56] OECD, Commission E . *Health at a Glance: Europe 2018* 2018.

[57] Tragakes E , Brigis G , Karaskevica J , et al. Health systems in transition: Latvia: health system review. Copenhagen: WHO Regional Office for Europe; 2008.

